# Heavy Foils

**Published:** 1871-07

**Authors:** A. A. Blount


### HEAVY FOILS.

BY A. A. BLOUNT, D. D. S.

Some time after heavy foils were discussed by experienced dentists, I made up my mind to give them a trial, and I am now satisfied from my own experience, that a much better and more perfect filling can be made with them, than with the lighter foils exclusively in use heretofore. I shall, in this connection, give my method of filling with heavy foils.

If the cavity is a small one, I commence the filling with No. 4 foil; if a large one, with No. 6, filling nearly to the surface, taking care to have the foil as level as possible to receive the heavy numbers. Take No. 60, cut the pieces a little larger than the surface of the filling, and "planish" down piece by piece with a "perfectly smooth" pointed instrument. Upon finishing the filling there will be no instrument marks upon the surface to mar its beauty. Frequently fillings made with the greatest care, with serrated points, and finished in the highest style of the art, present the appearance of not having been finished at all. Another advantage of finishing with planishing points, is a more perfect adaptation of the foil to the edges of the cavity, for the reason that a perfectly level smooth point, not only condenses, but at the same time, spreads the foil,—the heavier the foil the more it will spread,—so that a latteral pressure is had that forces the foil against the edges of the cavity, and makes the filling perfectly secure. With a serrated point, the pressure is in a direct line with the instrument, and only spreads the foil in proportion as the serrations are wedge shaped. While condensing a pellet, a tendency to curl up may have been observed, and sometimes in condensing a filling in the center, it will draw away from the edges of the cavity. That I should say is entirely owing to the deep serrated points, the more shallow and finer the serrations, the less the tendency to curl. Consequently with a smooth point, the difficulty would be entirely obviated.

In building up a tooth, I have often put from four to six pieces of No. 60 foil together, and planished them all down at one time, the foil working with the same facility as a single piece.

In building out the incisors the smooth points can be worked to the best advantage. When the walls of the cavity are frail and friable, the least touch of a sharp serrated instrument would shatter it, whereas with a smooth point, the foil can be laid over the edges of the frailest enamel, and worked without the fear of chipping off the thinnest edge.

I have put fillings, built out to restore the coutour of the tooth, that have not been touched with any but the smooth point, to the severest test, to ascertain if the adhesion is perfect, and am fully satisfied that the adhesion of the gold is much more perfect than when the serrated points are used. The satisfaction that one experiences in working the gold with planishing points, is calculated to entirely relieve the mind of anxiety in regard to the finish of the filling, or the fracture of the tooth. If the foil is too high in one place and too low in another, I planish it down and draw it towards the low place. If at any point the foil does not lay close to the edge of the cavity, instead of taking the risk of cracking the edge of the enamel with the instrument, by wedging in a small piece of gold, commence back towards the center of the filling, and spread the gold in the direction desired.

In fact, the gold can be drawn or otherwise shaped, as one would shape a piece of metal with a smooth hammer upon an anvil. I have shaped a few instruments to enable me to more fully test this method of manipulating gold, and am so fully satisfied of its superiority over any other method, that I am willing to use none other than smooth points. I have filled cavities no larger than a pins head, with No. 60 foil, and then tried to pry it out, which I found could be done only by chipping it piece by piece. The gold seemed to be driven into all the inequalities of the cavity, even the smallest scratch of the excavator was filled. By using the magnifying glass, it seemed as though the gold was driven into the substance of the tooth itself. And I have filled large cavities which took an eighth ounce of gold, No. 60, with but a single instrument, in one-third less time than it would take by the use of serrated instruments.

I am satisfied hereafter to use no other points than smooth ones, and no foil lighter than No. 10, and as much heavier as I can get; but I believe No. 60 will answer for all practical purposes, as we can make it as heavy as desired by putting piece upon piece. The way I do that is to anneal each piece separately, and lay them together, and then anneal the whole mass. I observe in working foil in this manner that the instrument adheres to it, as though it had some sticky substance upon it. I do not know what produces this effect, unless it is electricity. It is not necessary to buy a new set of instruments to make the experiment of filling a tooth in this manner, you have only to take an excavator, and file the end off smooth and polish it well to make the trial, and then select an incisor that requires building out, which will present the most favorable case for the experiment.

I am satisfied after the first trial you will not abandon the planishing points, for the more you work gold with them, the greater will be your satisfaction and pleasure.

